# Mechanisms of Light and Music Stimulation Controlled by a Person’s own Brain and Heart Biopotentials or Those of another Person

**DOI:** 10.17691/stm2020.12.4.03

**Published:** 2020-08-27

**Authors:** A.I. Fedotchev, S.B. Parin, L.V. Savchuk, S.A. Polevaya

**Affiliations:** Leading Researcher, Laboratory of Reception Mechanisms; Institute of Cell Biophysics, Russian Academy of Sciences, 3 Institutskaya St., Pushchino, Moscow Region, 142290, Russia; Professor, Department of Psychophysiology ; National Research Lobachevsky State University of Nizhni Novgorod, 23 Prospekt Gagarina, Nizhny Novgorod, 603950, Russia; PhD Student; National Research Lobachevsky State University of Nizhni Novgorod, 23 Prospekt Gagarina, Nizhny Novgorod, 603950, Russia; Head of the Department of Psychophysiology National Research Lobachevsky State University of Nizhni Novgorod, 23 Prospekt Gagarina, Nizhny Novgorod, 603950, Russia

**Keywords:** audio-visual stimulation, exposure to light and music, EEG, heart rate, one’s own–another person’s biopotentials, interoceptive signals, correction of stress-induced states.

## Abstract

**The aim of the study** was to carry out comparative analysis of effects observed in subjects exposed to light and music stimulation controlled by their own brain and heart biopotentials (closed-loop method) or biopotentials of another person.

**Materials and Methods.:**

Volunteers under stress participated in two experiments in pairs. In the first experiment, light and music stimulation effects formed in each subject in a pair on the basis of their own brain and heart biopotentials, while in the second experiment, they formed on the basis of biopotentials of the other subject.

**Results.:**

Both types of exposure caused reducing the tension of the regulatory systems in the body, reducing stress levels and improving the emotional state due to the mechanisms of multisensory integration and neuroplasticity. A significant increase in the power of the main EEG rhythms, accompanied by significant positive changes in psychological testing results and positive emotional responses to stimulation was observed only during light and music stimulation controlled by the subjects’ own brain and heart biopotentials. These data are attributable to the integration of perception and processing of interoceptive signals significant for humans into the resonance mechanisms of the central nervous system, providing normalization of functional state due to stimulation.

**Conclusion.:**

The data obtained can be used for developing the effective methods of personalized light and music stimulation aimed at timely elimination of functional disorders and returning the human body to homeostasis.

## Introduction

Audio-visual stimulation (AVS) has recently attracted increased attention due to a number of advantages: mobility, ease of implementation, fast and complex effects, capability to improve human health and functional state [1–6]. Recent studies have demonstrated the possibility of using AVS to improve sports training regimes [7–9] and to confirm the differential diagnosis in chronic disorder of consciousness [10]. In attempt to identify AVS mechanisms, we evaluated the efficacy of using a single AVS session to correct the functional state of the body [11], studied the effects of various AVS types [12], including music-light effects formed on the basis of human bioelectrical processes [13].

Earlier [14], we carried out a comparative analysis of effects observed when stress-induced states were suppressed by light-music stimulation with and without control feedback signals from subjects’ brain and heart biopotentials. It was found that the most remarkable shifts in objective and subjective parameters, including the maximum increase in EEG alpha power as compared to the background, positive emotional response and shifts in the functional state of the body were noted in cases when AVS was controlled by directly recorded the subjects’ own electrophysiological characteristics. These and our previously obtained data [15] suggest that the revealed effects are attributable to the involvement of perception and processing of interoceptive signals significant for humans in the mechanisms of multisensory integration, neuroplasticity, and brain resonance mechanisms providing normalization of the functional state under the influence of AVS.

Experimental verification of this assumption can be carried out in strictly controlled studies, where light and music effects controlled by biopotentials of another person will serve as control. When the processes of perception and processing of the subjects’ own interoceptive signals are excluded, such control allows for more detailed analysis of the mechanisms of action.

**The aim of the study** was to reveal the mechanisms of AVS performing the comparative analysis of the effects observed in subjects exposed to light and music stimulation controlled by their own brain and heart biopotentials (closed-loop method) or biopotentials of another person.

## Materials and Methods

The study involved 30 subjects aged 18 to 23 years, students of National Research Lobachevsky State University of Nizhni Novgorod, who were under stress during the examination session.

Subjects divided into teams of two volunteered to participate in two experiments (examinations) carried out with a time interval of 1–2 days. In the first experiment, the effects of stimulation with light and music presented to each subject in the pair were formed on the basis of their own brain and heart biopotentials, while in the second experiment, they formed on the basis of biopotentials of the other subject.

The study was carried out in accordance with the Helsinki Declaration (2013) and approved by the Ethics Committee of National Research Lobachevsky State University of Nizhni Novgorod. Written informed consent was obtained from each subject and signed by them after being informed about the experimental procedures, the potential risks, and benefits of participation.

To assess the psychophysiological state of the subjects, they underwent questioning and initial testing at the beginning of each experiment using two previously approved [16] tests:

1. WAM test, in which the subjects gave an assessment of their current wellbeing, activity, and mood;
2. EDL test, which allows determining the current level of emotional disadaptation of a person.

The subjects were examined in pairs, using two parallel experimental facilities. After the initial testing, the EEG electrodes were placed (the active electrode in the Cz lead, the reference and the ground electrodes on the earlobes). The original electrocardiogram recording system with online analysis of heart rate variability indicators [17], as well as Philips SBC HL140 stereo headphones (Netherlands) and darkened-lens goggles with built-in red LEDs of power limited to 100 μW were employed. The goggles and stereo headphones of both subjects were connected to one of the computers. The participants were told to sit still with their eyes closed during all examinations.

Each experiment began with a 30-second recording of the background electrical activity of the brain with EEG filtering range of 2–32 Hz and signal sampling frequency of 100 Hz. During the recording, the narrow-frequency (0.4–0.6 Hz) spectral component dominant in the examined subject was determined in EEG alpha range (8–13 Hz) using the original modification of dynamic spectral analysis based on fast Fourier transforms [18].

Next, the experimental facility connected to the glasses and headphones of both subjects was switched on in the operating mode for 10 min, during which time the subjects were exposed to light and music effects formed on the basis of brain and heart biopotentials of one of them. During this procedure, the current amplitude of the detected EEG oscillator was converted into music-like signals resembling flute sounds in tone quality and smoothly varying in pitch (the range of 100–2000 Hz) and intensity (the range of 0–40 dB), in direct correlation with the current amplitude of EEG oscillator. These music-like stimuli generated on the basis of EEG signal were supplemented by weak (nearly 10 dB) sound signals generated by electrocardiogram recording system and corresponding to the current heart rate of the subject. At the same time, the subject was exposed to LED stimuli in strict accordance with the current values of their native EEG. To achieve this, the digitized EEG values were regulated with the largest negative value of EEG signal corresponding to the minimum LED luminescence and the largest positive value corresponding to the maximum.

After stimulation, recording of EEG and cardio intervals was continued for 2 min to measure the aftereffects, the subjects were again tested and interviewed about their subjective sensations during the sessions.

The second examination was similar to the first one, but the subjects changed places at the experimental facilities and the light-music stimulation effects were formed on the basis of brain and heart biopotentials of the second subject in the pair.

The power parameters of the EEG theta, alpha, beta rhythms, cardiovascular system parameters, and the results of WAM and EDL tests were analyzed when processing the results. To record and analyze the cardiac rhythm, the technology of event-related telemetry of heart rhythm was used [19]. The sequence of R–R intervals of the ECG was transmitted from the miniature sensor platform ZephyrTM HxMTM Smart — Zephyr BIO PACH BH3-M1 (Zephyr Technology, USA), mounted on the subjects’ chests, to the smartphone via Bluetooth. After processing, the data were transmitted to the dedicated server system in the Internet via GSM channels. The processing algorithm consisted of the following steps: fragmentation of the received R–R signal with a time window of 100 s and a time shift of 10 s; calculation of the frequency spectrum by the method of non-uniform discrete Fourier transform of signals for the obtained windows; dividing the spectrum into ranges (VLF — 0.003–0.040   Hz, LF — 0.04–0.15   Hz, and HF — 0.15–0.4 Hz); at the last stage, calculating such derivatives as the total power of heart rate variability (HRV) spectrum and sympathovagal balance (vegetative balance index) — LF/HF.

The main attention in the analysis of HRV was paid to the duration of the R–R intervals, the total power of the HRV spectrum as a reflection of the adaptive potential of the central nervous system and the vegetative balance index as an indicator of tension of regulatory systems.

**Statistical processing** of the results was carried out using the SigmaPlot 11.0 software package. To assess the normality of distribution, Shapiro–Wilk test was used. Since all samples corresponded to the normal distribution as the main characteristics, the arithmetic mean (M) and standard errors (m) were calculated. The differences between the samples were estimated using the paired Student t-test to determine shifts (signed) of parameters as compared to the background and to assess the significance levels of these shifts. Differences were considered statistically significant at p≤0.05.

## Results

**Figure 1:**
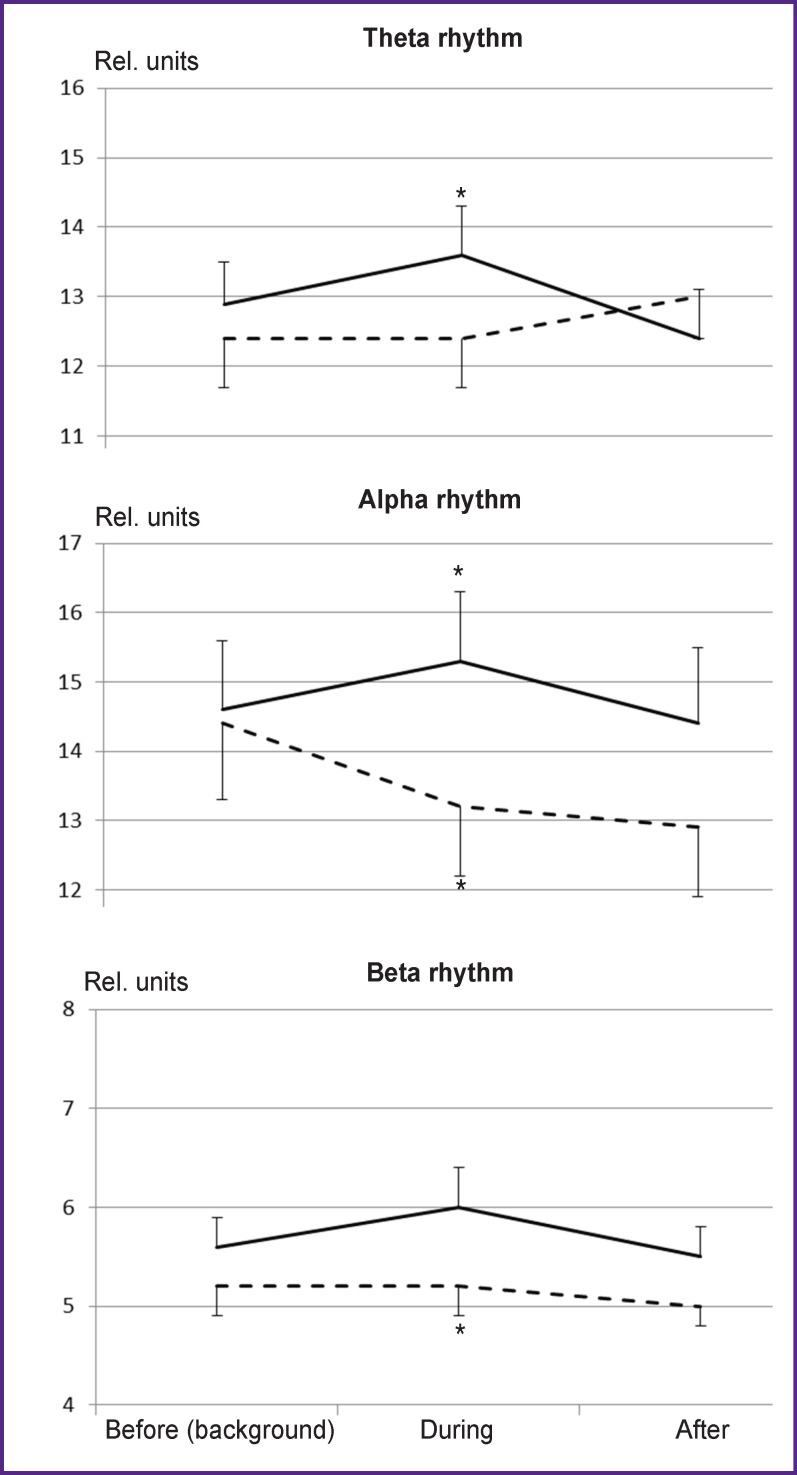
Power dynamics (rel. units) of EEG theta, alpha, and beta rhythms in experiments using light and music stimulation controlled by the subject’s own (*full line*) or another person’s (*dotted line*) biopotentials. Asterisks indicate statistically significant (p<0.05) differences in parameters during the stimulation as compared to the background

EEG effects were evaluated by comparing the dynamics of the EEG rhythms before, during, and after each type of exposure (Figure 1). Multidirectional dynamics of EEG rhythm intensity was established with two types of stimulation. There was a statistically significant increase in the power of all EEG rhythms when light and music stimulation effects were controlled by the subjects’ own brain and heart biopotentials. However, the use of other people’s biopotentials did not lead to changes in the power of the theta and beta rhythms or, on the contrary, statistically significantly reduced EEG alpha rhythm intensity.

**Figure 2:**
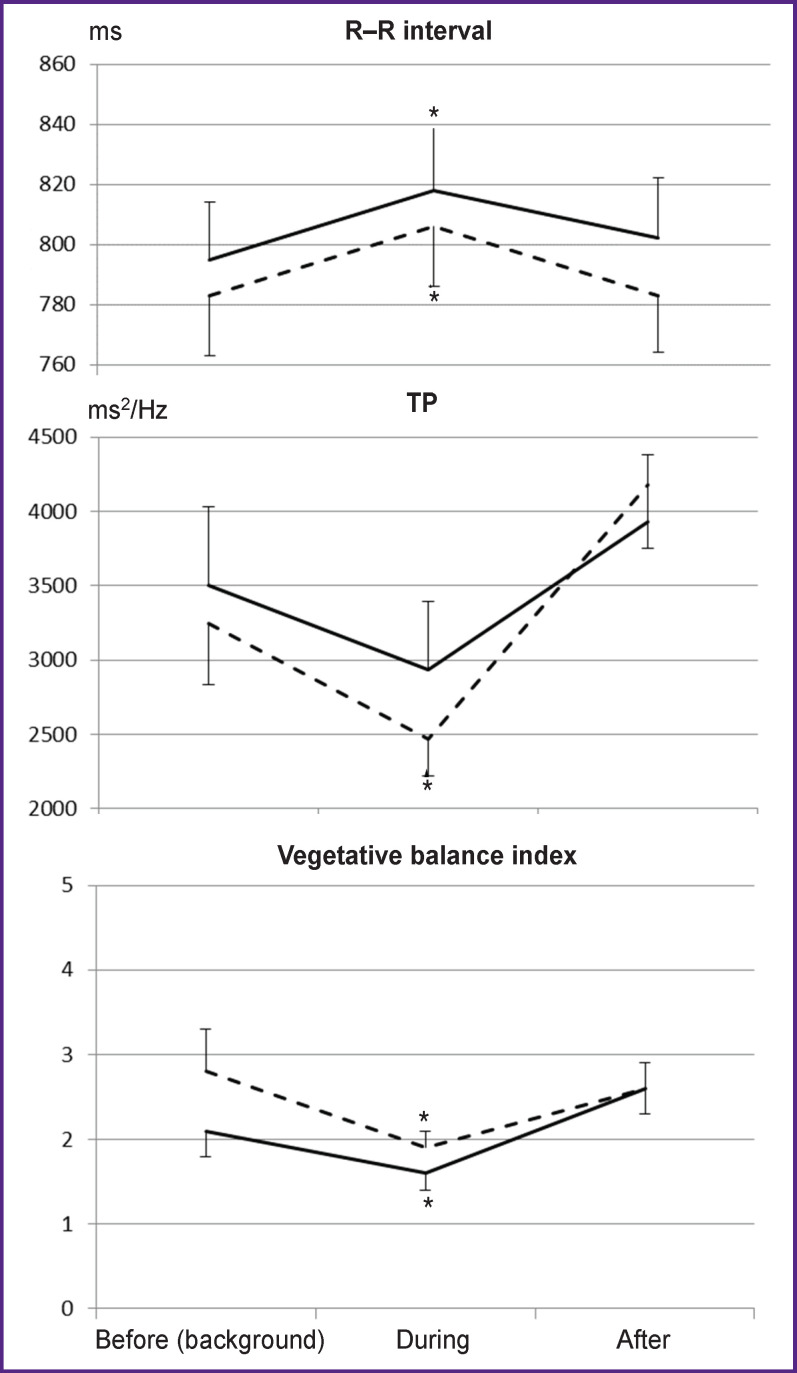
The dynamics of R–R intervals, the total power of the HRV spectrum (TP), and the vegetative balance index in experiments using light and music stimulation controlled by the subject’s own (*full line*) or another person’s (*dotted line*) biopotentials. Asterisks indicate statistically significant (p<0.05) differences in parameters during the stimulation as compared to the background

The dynamics of cardiovascular activity parameters was analyzed in a similar way (Figure 2). The figure shows that both types of stimulation make the body pass into a low-energy state with a decrease in stress parameters of regulatory systems: the duration of cardio intervals increases statistically significantly, and the vegetative balance index decreases. Under the influence of stimulation controlled by another person’s biopotentials, there is a statistically significant decrease in the total power of the HRV spectrum, indicating rigidity of the heart rhythm and a decrease in the adaptive potential of the central nervous system.

Significant differences were also observed in the subjective reactions of the tested persons to the presented stimuli. There were shifts in the results of WAM and EDL tests under the influence of each type of stimulation as compared to the initial level. The data obtained are given in the table, making it clear that under the influence of light-music stimulation based on the subjects’ own brain and heart biopotentials, there is a statistically significant increase in the subjects’ wellbeing values and a statistically significant decrease in activity values and emotional disadaptation level, pointing to the relaxation effect of stimulation. When using control signals from other subjects’ biopotentials, no significant changes in the tested parameters were detected.

**Table:** Shifts in parameters as a result of light and music stimulation controlled by the subject’s own and another person’s biopotentials and the level of significance of these shifts

Parameter (points)	Biopotentials			
	the subject’s own		another person’s	
	Shift (М±m)	p	Shift (М±m)	p
WAM test, wellbeing	**0.20±0.09**	**0.032**	–0.01±0.16	0.922
WAM test, activity	**–0.31±0.13**	**0.027**	–0.25±0.20	0.228
WAM test, mood	0.13±0.11	0.226	0.01±0.11	0.953
EDL test, emotional disadaptation level	**–0.29±0.14**	**0.044**	–0.12±0.15	0.275

Note: Statistically significant shifts are in bold (p<0.05).

Questioning of the subjects about their subjective sensations during the experiments revealed positive attitude to the treatment sessions, reduction of stress level, and improvement of emotional state. Experiments with the use of control signals from the subjects’ own biopotentials were perceived even more emotionally. Most of the subjects (23 out of 30) found such exposure pleasant and soothing.

## Discussion

Common as well as specific features of the two stimulation types were identified in the conducted controlled study where each subject was a source of control signals for both himself and another person. Heart responses to stimulation were common, demonstrating a decrease in the tension in the body’s regulatory systems, as well as positive subjective reactions to the sessions, a decrease in stress levels, and improvement of the emotional state. Such similarity is most likely based on multisensory integration mechanisms [15, 20] and neuroplasticity mechanisms [21].

The main difference between reactions to the two stimulation types was manifested in a statistically significant increase in the power of the main EEG rhythms when using the subject’s own biopotentials and in the absence of such effects when using another person’s biopotentials. A significant increase in the intensity of all EEG rhythms during light and music stimulation, controlled by the subject’s own biopotentials, directly indicates the participation of resonance mechanisms of brain activity in these effects [22]. According to modern concepts of AVS mechanisms, synchronism of stimulation rhythms and the frequencies of endogenous vibrational neurodynamic processes in the central nervous system can lead to resonance phenomena and, therefore, to synchronization of previously uncorrelated sources of spontaneous brain rhythms [23]. This synchronism is provided when generating rhythmic light and music effects based on the current values of the subject’s own biopotentials. When using control signals from another person’s biopotentials, the synchronism does not occur, moreover, suppression of spontaneous brain rhythms is observed at the frequency of the EEG alpha rhythm.

The study revealed other differences in responses to the two types of stimulation. Light-music stimulation, controlled by the subject’s own biopotentials, leads to positive shifts in psychological testing scores and positive emotional responses to stimulation. When using another person’s biopotentials, there are no such effects. Moreover, changes occur in cardiac activity, indicating a decrease in the adaptive potential of the central nervous system.

When explaining the above differences, it should be borne in mind that brain and heart biopotentials are a source of interoceptive signals, which, according to modern concepts, play an important role in maintaining the optimal physical, emotional, and mental health of a person [24]. The use of interoceptive signals about the state of a person’s own body in biofeedback procedures is considered a promising area of modern research requiring in-depth analysis [25]. Our data on the presence of various positive effects during light and music stimulation controlled by the subjects’ own biopotentials and the absence of such effects when using other persons’ biopotentials complement and expand these ideas.

## Conclusion

The closed-loop method [26], which involves the use of a person’s own bioelectric processes in the organization of therapeutic intervention, is a promising way of developing biomedical technologies [27]. Therefore, improvement of mechanisms that determine the effects of using one’s own or another person’s biopotentials as a factor of online modulation of light and music stimulation seems to be an urgent task.

The conducted study confirmed and refined the previous hypothesis [14] about the participation of multisensory integration mechanisms, neuroplasticity, and resonance brain mechanisms in light-music stimulation effects automatically generated by the subject’s brain and heart biopotentials. The main role in the positive response of the body to the stimulation is played by the simultaneous involvement of perception and processing of significant interceptive signals and resonance mechanisms of brain activity.

The data obtained can be used for developing the effective methods of personalized light and music stimulation aimed at timely elimination of functional disorders and returning the human body to homeostasis.
